# Case Study of the Response of N^6^-Methyladenine DNA Modification to Environmental Stressors in the Unicellular Eukaryote Tetrahymena thermophila

**DOI:** 10.1128/mSphere.01208-20

**Published:** 2021-05-28

**Authors:** Yalan Sheng, Bo Pan, Fan Wei, Yuanyuan Wang, Shan Gao

**Affiliations:** a Institute of Evolution & Marine Biodiversity, Ocean University of China, Qingdao, China; b Laboratory for Marine Biology and Biotechnology, Qingdao National Laboratory for Marine Science and Technology, Qingdao, China; c MOE Key Laboratory of Marine Genetics and Breeding, College of Marine Life Sciences, Ocean University of China, Qingdao, China; Academia Sinica

**Keywords:** 6mA, starvation, unicellular eukaryote, *Tetrahymena thermophila*

## Abstract

Rediscovered as a potential epigenetic mark, N^6^-methyladenine DNA modification (6mA) was recently reported to be sensitive to environmental stressors in several multicellular eukaryotes. As 6mA distribution and function differ significantly in multicellular and unicellular organisms, whether and how 6mA in unicellular eukaryotes responds to environmental stress remains elusive. Here, we characterized the dynamic changes of 6mA under starvation in the unicellular model organism Tetrahymena thermophila. Single-molecule, real-time (SMRT) sequencing reveals that DNA 6mA levels in starved cells are significantly reduced, especially symmetric 6mA, compared to those in vegetatively growing cells. Despite a global 6mA reduction, the fraction of asymmetric 6mA with a high methylation level was increased, which might be the driving force for stronger nucleosome positioning in starved cells. Starvation affects expression of many metabolism-related genes, the expression level change of which is associated with the amount of 6mA change, thereby linking 6mA with global transcription and starvation adaptation. The reduction of symmetric 6mA and the increase of asymmetric 6mA coincide with the downregulation of AMT1 and upregulation of AMT2 and AMT5, which are supposedly the MT-A70 methyltransferases required for symmetric and asymmetric 6mA, respectively. These results demonstrated that a regulated 6mA response to environmental cues is evolutionarily conserved in eukaryotes.

**IMPORTANCE** Increasing evidence indicated that 6mA could respond to environmental stressors in multicellular eukaryotes. As 6mA distribution and function differ significantly in multicellular and unicellular organisms, whether and how 6mA in unicellular eukaryotes responds to environmental stress remains elusive. In the present work, we characterized the dynamic changes of 6mA under starvation in the unicellular model organism Tetrahymena thermophila. Our results provide insights into how *Tetrahymena* fine-tunes its 6mA level and composition upon starvation, suggesting that a regulated 6mA response to environmental cues is evolutionarily conserved in eukaryotes.

## INTRODUCTION

N^6^-methyladenine DNA modification (6mA) was recently reported to act as a potential epigenetic mark sensitive to environmental stressors. In human cell lines, mitochondrial DNA 6mA levels increase significantly under hypoxic stress (1). In mouse brain, 6mA levels increase dramatically in response to chronic stress and is inversely associated with stress-response neuronal genes (2). In Caenorhabditis elegans, 6mA is upregulated under conditions of mitochondrial stress, a response that is essential for the transmission of stress adaptation to progeny (3). In rice, 6mA levels negatively correlate with cold tolerance and positively correlate with salt and heat tolerance (4). All these findings suggest a process of active 6mA regulation for stress adaptation in multicellular eukaryotes.

It should be noted, however, that 6mA in unicellular eukaryotes displays distinctly different characteristics than its counterparts in multicellular organisms. While unicellular 6mA is preferentially located in ApT dinucleotides and is associated with actively transcribed genes, the opposite is true for multicellular 6mA (5–10). More intriguingly, these modifications are catalyzed by two divergent groups of MT-A70 methyltransferases (MTases), AMT1 and METTL4, respectively (5, 11–13). These discrepancies prompted us to investigate whether and how 6mA in unicellular eukaryotes responds to environmental stress.

6mA in the unicellular model organism Tetrahymena thermophila was detected decades ago (14, 15) and is now the subject of intensive study due to the recent resurgence of interest in it (5, 12, 16–18). Upon starvation conditions, *Tetrahymena* cells undergo oral replacement, transformation into fast-swimming dispersal forms, and downregulation of cell size (19, 20). Starvation is not only a distinct physiological state but also a prerequisite to induce sexual reproduction (21–24). During this process, the anterior cell cortex is remodeled in preparation for conjugation. This involves novel membrane synthesis and glycoprotein “capping,” otherwise known as tip transformation (25–28). These ultrastructural and biochemical changes all occur in a relatively short time and involve epigenetic factors. Indeed, the phosphorylation state of H1 was reported to regulate genes expression in response to starvation (29).

In this study, we applied single-molecule, real-time (SMRT) sequencing to investigate how 6mA level and composition in T. thermophila are affected by starvation. We linked the 6mA change with the gene expression level change and physiological events that occur during starvation. We also attributed 6mA dynamic shift to the coincidental change of several MT-A70 6mA methyltransferases.

## RESULTS

### DNA 6mA level in *Tetrahymena* was reduced during starvation.

**FIG 1:**
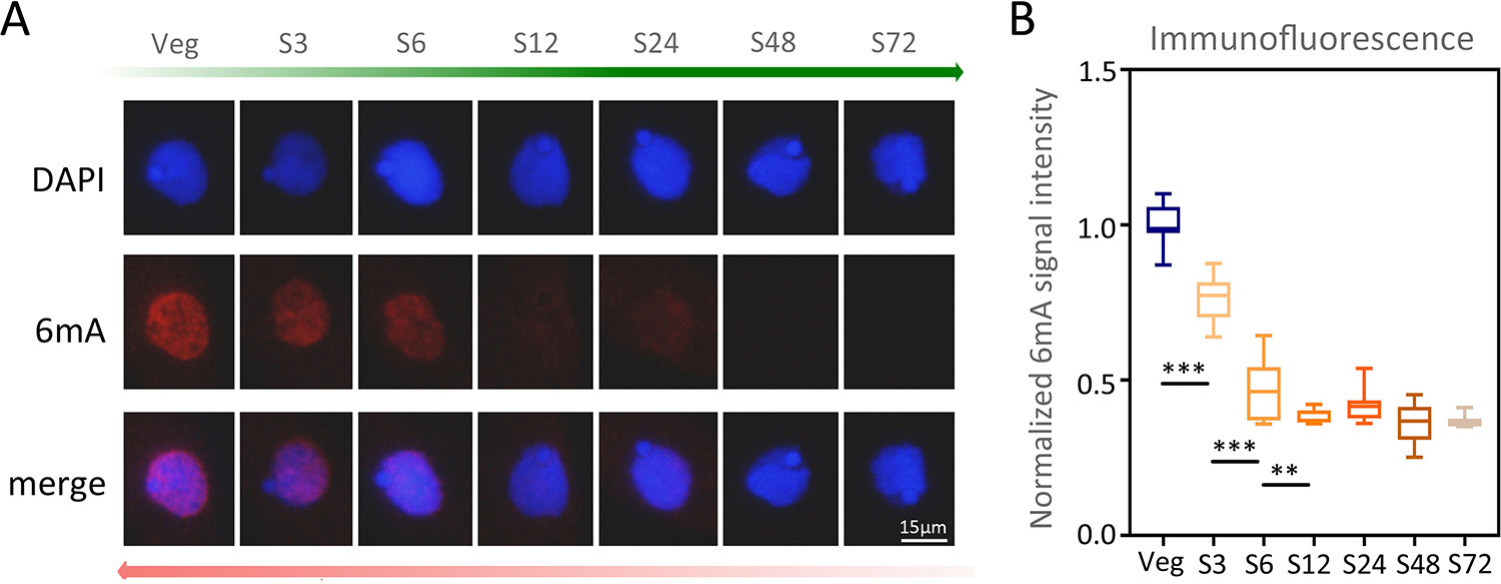
DNA 6mA level was dramatically reduced during starvation. (A) IF staining of DNA 6mA in logarithmically vegetative (Veg) and starved cells. S3 to S72 represent 3 to 72 h after starvation. (B) Statistical analysis of 6mA IF signal intensity in panel A. Cell images were processed by ImageJ (Veg, *n* = 112; S3, *n* = 111; S6, *n* = 111; S12, *n* = 105; S24, *n* = 112; S48, *n* = 105; S72, *n* = 109). Data are presented as box plots (from top to bottom: maximum, first quartile, median, third quartile, and minimum). Student's *t* test was performed (***, *P* < 0.001; **, *P* < 0.01). Note that there was no significant difference in cells starved for 12 to 72 h.

To detect whether 6mA in *Tetrahymena* was affected by starvation, we performed immunofluorescence (IF) staining of vegetative and starved cells with a 6mA-specific antibody. The 6mA level in the macronucleus (MAC) dropped dramatically after only 3 h starvation, and it was further reduced with the progression of starvation (Fig. 1A and B), while the MAC DNA content remained stable (see Fig. S1 in the supplemental material) (30, 31).

10.1128/mSphere.01208-20.1 FIG S1 ImageJ statistical analysis of DAPI signal intensity in vegetative and starved cells (Veg, *n* = 112; S24, *n* = 112). Download FIG S1, PDF file, 0.2 MB. Copyright © 2021 Sheng et al. 2021 Sheng et al. https://creativecommons.org/licenses/by/4.0/ This content is distributed under the terms of the Creative Commons Attribution 4.0 International license.

**FIG 2:**
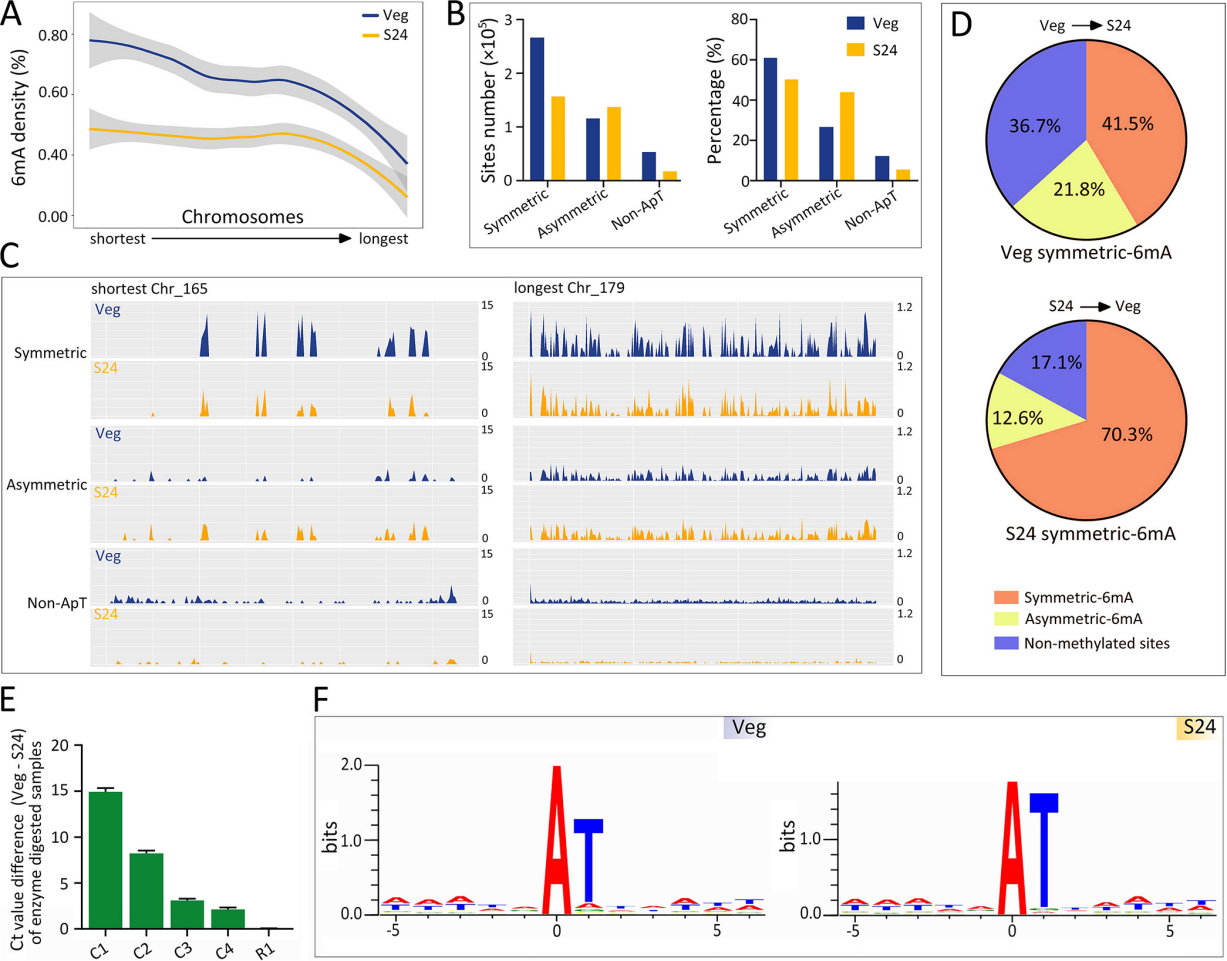
The genome-wide distribution of 6mA was affected in starved cells. (A) 6mA density (6mA/A) is dramatically reduced in starved cells (orange) compared to vegetative cells (blue) across the 180 non-rDNA chromosomes. Chromosomes are arranged by length, from shortest to longest. (B) Classification of 6mA sites according to their sequence preference in vegetative (blue) and starved (orange) cells. The left and right panels represent 6mA site number and percentage (a particular class of 6mA/all 6mA), respectively. See Table 1 for details. (C) 6mA distribution across the shortest (left) and longest (right) chromosomes. The height of lines represents the 6mA density (percent). Note that the 6mA density of the shortest chromosome is much greater than that of the longest chromosome. (D) Overall behaviors of individual 6mA sites in vegetative and starved cells, showing results from SMRT sequencing data. (E) The methylation status change of selected conversion sites (C1 to C4; symmetric in Veg cells and unmethylated in S24 cells) and retain site (R1; symmetric in both Veg and S24 cells) was confirmed by DpnI/DpnII digestion and qPCR analysis. The Ct value difference (Veg − S24) of conversion sites was larger than zero, while that of the retain site was close to zero. (F) Sequence logos for 6mA (at position 0) in vegetative and starved cells.

We next performed SMRT sequencing of cells starved for 24 h (S24). In total 1,077,887 reads were generated, corresponding to 103× average coverage of the *Tetrahymena* MAC genome (Table S1). We called 312,521 sites with high confidence (normalized coverage > 25×; quality value [Qv] > 30), representing 0.39% of the total adenines (Table 1). This number is lower than that in vegetative (Veg) cells (0.54%; 436,276 sites) (Table 1) (12). 6mA density was evenly reduced across 180 non-rDNA chromosomes in starved cells relative to vegetative cells (Fig. 2A). Intriguingly, there was a negative correlation between 6mA density and chromosomal length (Fig. 2A). From the longest to the shortest chromosomes, 6mA density ranged from 0.18% to 1.45% in vegetative cells and from 0.12% to 0.93% in starved cells.

**TABLE 1:** Comparison of 6mA in vegetative and starved cells

6mA	Veg		S24	
	No. of sites	%	No. of sites	%
6mA density (6mA/A)		0.54		0.39
Methylated adenines	436,276		312,521	
Symmetric	133,199 × 2	61.1	78,507 × 2	50.3
**Asymmetric**	**116,465**	**26.7**	**137,926**	**44.0**
Non-ApT	53,413	12.2	17,581	5.6
L1 (0–20%)	1,214	0.3	359	0.1
L2 (20–84%)	14,649	3.4	2,459	0.8
L3 (40–60%)	66,749	15.3	19,300	6.2
L4 (60–80%)	191,161	43.8	116,167	37.2
**L5 (80–100%)**	**162,503**	**37.2**	**174,236**	**55.8**

10.1128/mSphere.01208-20.5 TABLE S1 SMRT sequencing data quality control. Download Table S1, DOCX file, 0.02 MB. Copyright © 2021 Sheng et al. 2021 Sheng et al. https://creativecommons.org/licenses/by/4.0/ This content is distributed under the terms of the Creative Commons Attribution 4.0 International license.

Together, these results demonstrated that 6mA level is globally reduced in *Tetrahymena* upon starvation.

### The percentage of highly methylated asymmetric 6mA was increased in starved cells.

Both the number and the percentage of symmetric 6mA and non-ApT 6mA were dramatically reduced in starved cells (Table 1; Fig. 2B, left). Despite the global reduction of 6mA level, the number (from 116,465 to 137,926) and the percentage (from 26.7% to 44.0%) of asymmetric 6mA were increased (Table 1; Fig. 2B). This phenomenon was illustrated in both the longest and shortest chromosomes, although there was a huge difference in the 6mA densities of these two chromosomes (Fig. 2C). To further explore this dynamic change, we traced the overall behavior of individual sites. It is clear that a large proportion of symmetric 6mA in vegetative cells was converted into asymmetric 6mA in starved cells (110,432 sites; 21.8% of vegetative symmetric 6mA), obviously exceeding the opposite trend (19,738 sites; 12.6% of S24 symmetric 6mA) (Fig. 2D). In addition, some symmetric 6mA in vegetative cells (97,836 sites; 36.7%) was unmethylated after starvation (Fig. 2D); this was independently verified by quantitative PCR (qPCR) analysis using DpnI- and DpnII-digested genomic DNA as templates (Fig. 2E). 6mA was further enriched at the sequence 5′-ApT-3′ in starved cells (ApT, 94.4%; non-ApT, 5.6%) compared to vegetative cells (ApT, 87.8%; non-ApT, 12.2%) (Table 1). Indeed, 6mA of starved cells showed higher preference for thymine (T) at the +1 position (Fig. 2F). This was largely due to the overcompensation between increased asymmetric 6mA and reduced symmetric 6mA.

**FIG 3:**
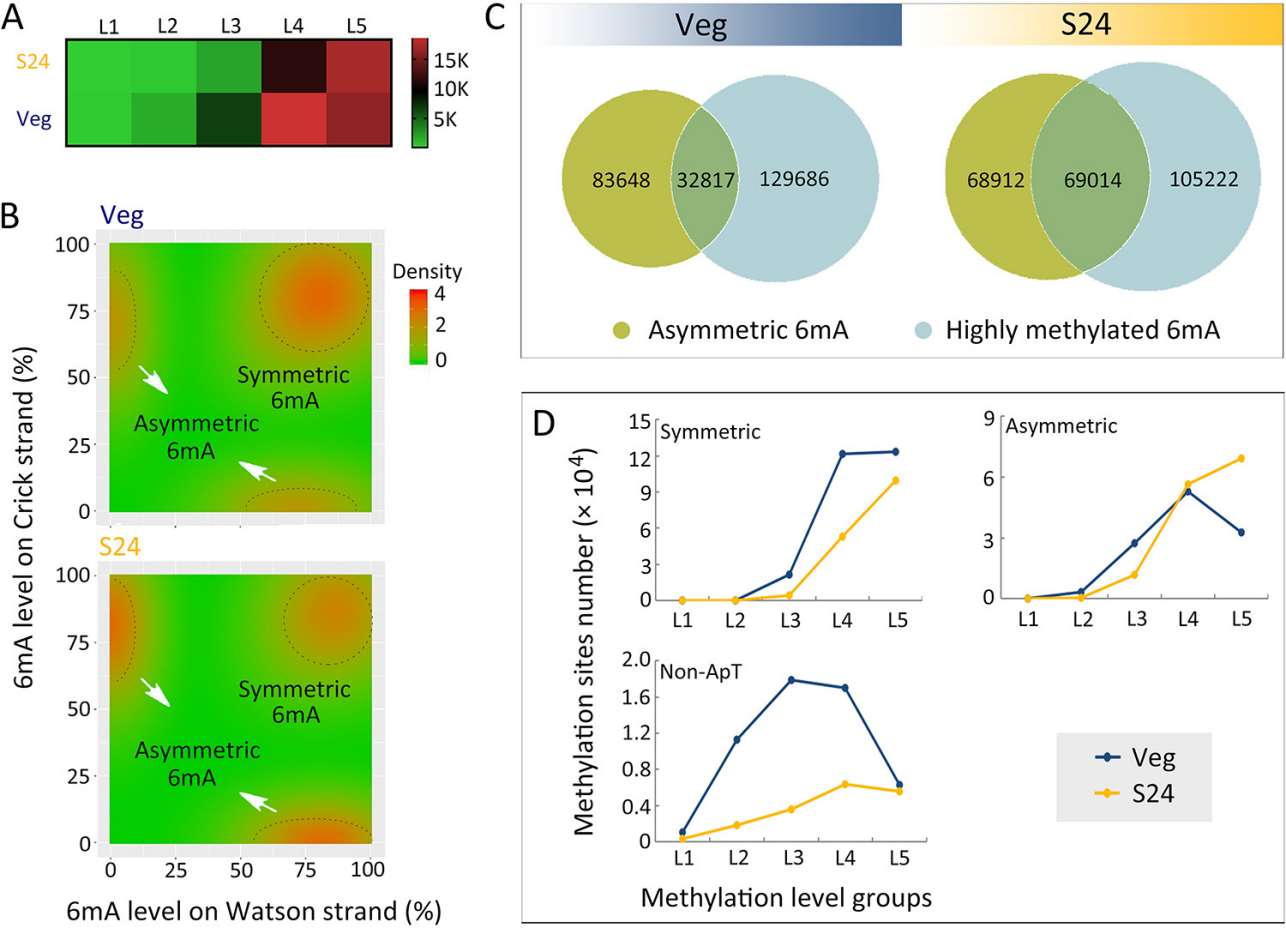
Methylation level of 6mA was dramatically changed in starved cells. (A) Classification of 6mA sites according to their methylation level (L1, 0 to 20%; L2, 20 to 40%; L3, 40 to 60%; L4, 60 to 80%; L5, 80 to 100%) in vegetative and starved cells. See Table 1 for details. (B) Density plots of 6mA distribution, according to methylation levels on Watson (*x* axes) or Crick (*y* axes) strands in vegetative (top) and starved (bottom) cells. Note that in starved cells, the highly methylated symmetric 6mA is decreased while the highly methylated asymmetric 6mA is increased. (C) Venn diagram of highly methylated 6mA (L5) and asymmetric 6mA showing that highly methylated asymmetric 6mA increased in starved cells, compared to vegetative cells. (D) Statistics of methylation level of symmetric, asymmetric and non-ApT 6mA. Only the number of the L5 asymmetric 6mA was doubled in starved cells compared to vegetative cells.

In the polyploid MAC, methylation levels varied almost continuously from 0 to 100 at different 6mA positions (8, 32–34). Thus, we divided the 6mA methylation level into five categories: L1 (0 to 20%), L2 (20 to 40%), L3 (40 to 60%), L4 (60 to 80%), and L5 (80 to 100%). 6mA number was increased only in the L5 category, while it was reduced in the other four categories (Table 1; Fig. 3A). To trace the contributors of the 6mA dynamic change, the distribution of methylated ApT sites was plotted against methylation levels. The peak corresponding to symmetric 6mA distribution was narrowed, reflecting the reduction of symmetric 6mA, and the asymmetric 6mA peaks shifted toward the terminal point, reflecting increased methylation levels (Fig. 3B). Consistent with this, we found that asymmetric 6mA is more closely linked with highly methylated 6mA in starved cells than in vegetative cells (22.1% versus 7.5% of total 6mA) (Fig. 3C), strongly suggesting that asymmetric 6mA in starved cells was driven to high methylation levels. In particular, only the number of the L5 asymmetric 6mA doubled in starved cells (Fig. 3D), being the main source for the increase of highly methylated sites.

Together, these results suggested that the proportion of asymmetric 6mA was increased in starved cells, most of which were also highly methylated.

### 6mA may be involved in gene regulation during starvation.

**FIG 4:**
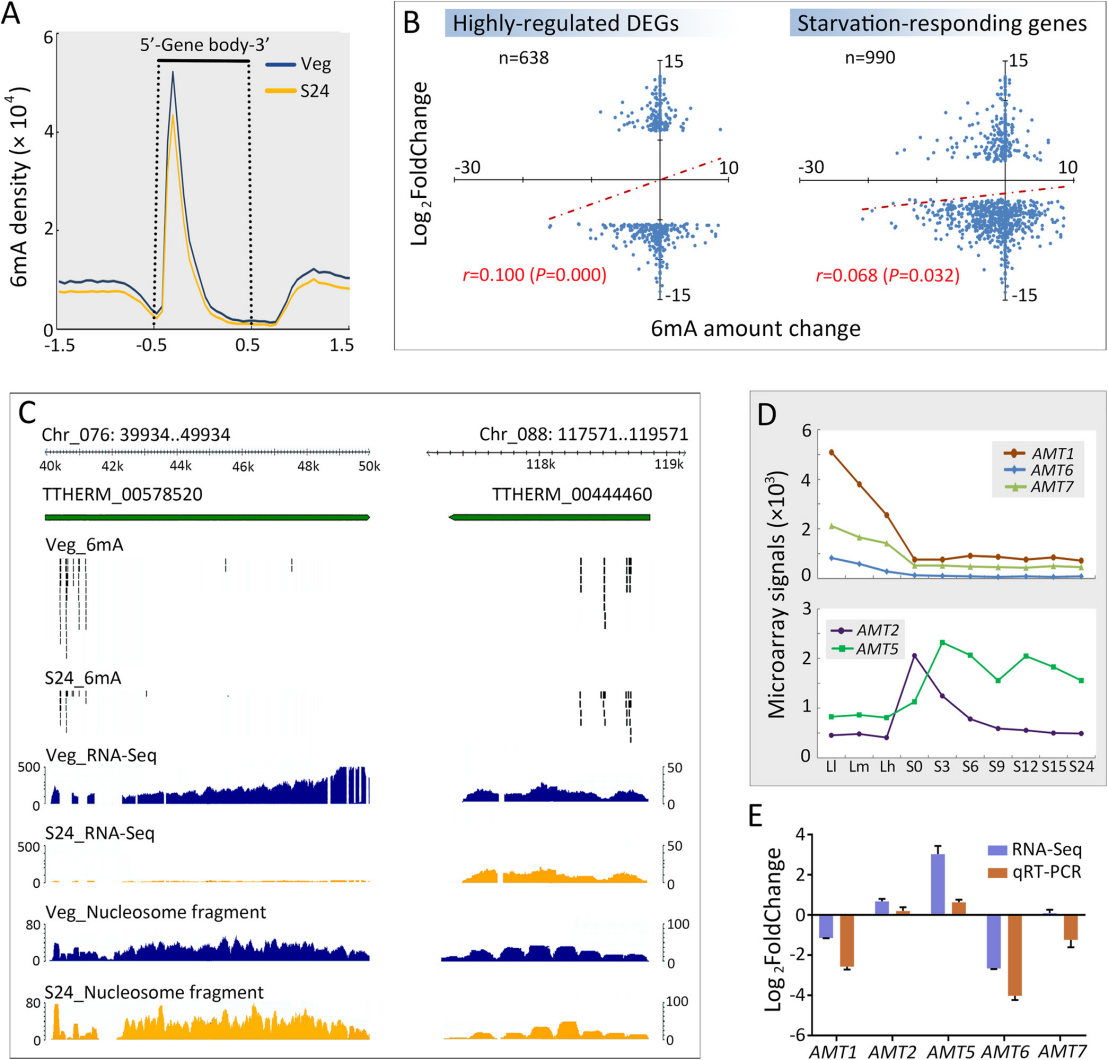
The increase/reduction of 6mA affected gene expression during starvation. (A) Composite analysis of 6mA distribution on the gene body of vegetative (blue) and starved (orange) cells. Genes were scaled to unit length and extended to each side by 1 unit length. Density was calculated as 6mA amount at a certain position/total 6mA amount. (B) The 6mA amount change (S24 − Veg) in the 1 kb downstream of TSS in highly regulated genes and starvation-responding genes presented a positive correlation with their expression level changes (log_2_ fold change). (C) GBrowse snapshot of two genes clearly showing that the expression level of the gene with 6mA reduction was significantly reduced in starved cells. (D) Expression levels of putative 6mA methyltransferases at different time points during vegetative growth and starvation, shown by microarray signals (59). For vegetative cells, Ll, Lm, and Lh correspond to ∼1 × 10^5^ cells/ml, ∼3.5 × 10^5^ cells/ml, and ∼1 × 10^6^ cells/ml, respectively. For starvation, ∼2 × 10^5^ cells/ml were collected at 0, 3, 6, 9, 12, 15, and 24 h, referred to as S0, S3, S6, S9, S12, S15, and S24. (E) Expression level change of putative 6mA methyltransferases, shown by both RNA-Seq and qRT-PCR analysis.

6mA was mapped to about 79.68% (20,923 genes; 377,600 sites) and 72.20% (18,960 genes; 275,956 sites) of polymerase II (Pol II)-transcribed genes in vegetative and starved cells, respectively. As reported previously (8, 12), 6mA was preferentially enriched at the 5′ end of the gene body in both vegetative and starved cells, although it showed a reduction in the latter (Fig. 4A).

We next explored whether the global reduction of 6mA affected gene expression, which was evaluated by steady-state transcriptome sequencing (RNA-Seq) levels. Indeed, a large number of differentially expressed genes (DEGs) were detected upon starvation (6,375 of 26,258 well-annotated genes), including 2,957 upregulated and 3,418 downregulated genes (*P*_adj_ < 0.05; log_2_ fold change < −2 or > 2) (Fig. S2A). The 6mA amount change (S24 − Veg) in the 1 kb downstream of transcription start sites (TSS) in highly upregulated and downregulated DEGs presented a positive correlation with their expression level changes (log_2_ fold change) (Fig. 4B). Notably, this weak yet significant correlation was also detected in starvation-responding (induced and repressed) genes (Fig. 4B). A GBrowse snapshot of two genes clearly showed that the expression level of one gene, which had 6mA reduction, was significantly reduced in starved cells, while that of the other gene, which had a minimal 6mA level change, was not affected (Fig. 4C). These results suggested that 6mA plays a potential role in gene expression regulation during starvation.

10.1128/mSphere.01208-20.2 FIG S2 A large number of DEGs were detected upon starvation. (A) Global transcriptome analysis in vegetative and starved cells. *P*_adj_ indicates the adjusted *P* value of the DEGs. (B) Gene ontology analysis of DEGs between vegetative and starved cells. Download FIG S2, PDF file, 1 MB. Copyright © 2021 Sheng et al. 2021 Sheng et al. https://creativecommons.org/licenses/by/4.0/ This content is distributed under the terms of the Creative Commons Attribution 4.0 International license.

Gene ontology analysis of differentially expressed genes revealed many conserved pathways affected by starvation, including phagosome, carbon metabolism, fatty acid metabolism, and oxidative phosphorylation (Fig. S2B), consistent with the physiological changes in starved cells (29, 35, 36). Intriguingly, several putative 6mA methyltransferases were also differentially expressed. While *AMT1* and its possible partners *AMT6*/*AMT7* (5, 12) were downregulated during starvation, the opposite was true for two other MT-A70 family members, i.e., *AMT2* and *AMT5* (12) (Fig. 4D and E; Table 2).

**TABLE 2:** Gene expression level change (Veg/S24) of methyltransferase genes

Gene	TTHERM no.	Log_2_ fold change	
		RNA-Seq	qRT-PCR
*AMT1*	00704040	−2.57	−1.15
*AMT2*	00388490	0.20	0.68
*AMT5*	00136470	0.64	3.03
*AMT6*	01005150	−4.02	−2.68
*AMT7*	00301770	−1.24	0.08

### Nucleosome positioning degree is increased in starved cells.

**FIG 5:**
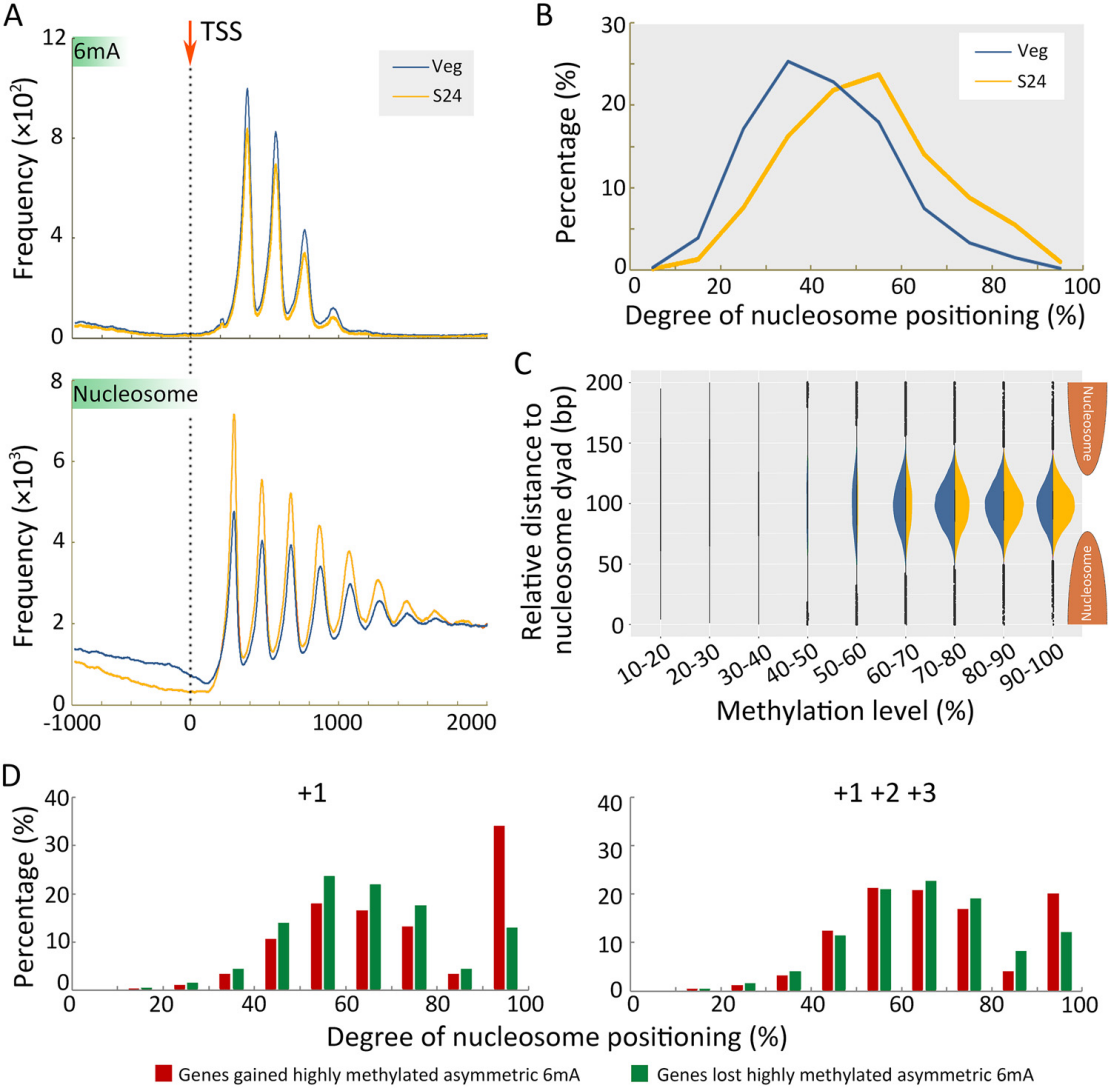
Nucleosome positioning was influenced by 6mA change in starved cells. (A) Distribution profiles of 6mA (top) and nucleosome (bottom) around TSS in vegetative (blue) and starved (orange) cells. (B) Nucleosome positioning degree in vegetative (blue) and starved (orange) cells. Degrees of positioning were calculated for the +1 to +5 nucleosomes. (C) 6mA distribution relative to the nucleosome dyad in vegetative (blue) and starved cells (orange). The violin plots show the density of 6mA between neighboring nucleosome dyads, grouped by methylation levels. Note that the highly methylated 6mA was increased in starved cells. (D) Nucleosome positioning degree of genes that gained (red) or lost (green) highly methylated (L5) asymmetric 6mA in the 1 kb downstream of the TSS in starved cells. Degrees of positioning were calculated for the +1 nucleosome (left) and +1, +2, and +3 nucleosomes (right).

Despite a slight global reduction, 6mA in starved cells was preferentially located in the linker DNA region (∼50 bp) between adjacent nucleosomes, as in vegetative cells (Fig. 5A). 6mA and nucleosomes displayed strong anticorrelation, showing two damped oscillations in opposite phases with the same periodicity (∼200 bp) downstream of TSS in both vegetative and starved cells (Fig. 5A). In starved cells, the amplitude (peak-to-trough distance) of nucleosome distribution was increased in spite of the reduced amplitude of 6mA distribution (Fig. 5A). Further analysis revealed a global increase in the degree of nucleosome positioning in starved cells, especially for +1, +2, and +3 nucleosomes (Fig. 5B; Fig. S3). We also discovered that although 6mA sites with intermediate methylation level (40 to 80%) in linker DNA were largely reduced, more highly methylated 6mA sites (90 to 100%) were found there (Fig. 5C). To further elaborate the role of 6mA in nucleosome positioning, we focused on genes that either gained or lost highly methylated (L5) asymmetric 6mA during starvation. The degree of nucleosome positioning was increased in the gain-of-6mA group, especially for +1 nucleosomes (Fig. 5D).

10.1128/mSphere.01208-20.3 FIG S3 Nucleosome positioning degree in vegetative (blue) and starved (orange) cells. Degrees of positioning were calculated for +1, +2, and +3 nucleosomes. Download FIG S3, PDF file, 0.6 MB. Copyright © 2021 Sheng et al. 2021 Sheng et al. https://creativecommons.org/licenses/by/4.0/ This content is distributed under the terms of the Creative Commons Attribution 4.0 International license.

## DISCUSSION

Increasing evidence indicates that DNA 6mA methylation could respond to environmental stressors in multicellular eukaryotes (2–4). But as multicellular and unicellular 6mA are different in terms of methylation amount, genomic distribution, catalyzing enzymes, and correlation with transcription (6, 8–10), how unicellular eukaryotes react in response to environmental cues via 6mA methylation is currently unknown.

In this study, we showed that the unicellular eukaryote T. thermophila responded to starvation by changing its 6mA methylation pattern: global 6mA level was largely reduced, especially for symmetric 6mA, contrasting with a dramatic increased level of 6mA in humans, mice, and C. elegans (1–3). It should be noted that the global reduction of 6mA level in starved cells was moderate, as shown by the SMRT sequencing data, while 6mA was almost undetectable after 3 h starvation, as shown by IF staining. As SMRT sequencing cannot distinguish 6mA from 6hmA (N^6^-hydroxymethyladenine), we suspected this discrepancy might be caused by 6mA conversion to 6hmA upon starvation, as reported in mammals (37). Without available antibody for 6hmA, we employed mass spectrometry analysis, in which 6hmA cannot be misinterpreted as 6mA due to their different molecular weights, to determine whether there is a peak representing 6hmA next to the 6mA peak. No such peak was detected in either vegetative or starved cells. The discrepancy between SMRT and IF results could be partially explained by the fact that starved cells undergo an increase in the size of condensed chromatin bodies (38), which may interfere with the antibody binding capacity. We also noticed that mass spectrometry analysis detected a minimal change of 6mA level in starved cells (Fig. S4). This slight difference between mass spectrometry and SMRT results may stem from different ways of processing data: mass spectrometry counted all detected methylated sites, while SMRT applied a strict cutoff for 6mA calling (Qv > 30; normalized coverage > 25×).

10.1128/mSphere.01208-20.4 FIG S4 Mass spectrometry analysis of 6mA, performed on three biological replicates for vegetative and starved cells. Data are presented as box plots. Student’s *t* test was performed. Note that there was no significant difference in vegetative and starved cells. Download FIG S4, PDF file, 0.3 MB. Copyright © 2021 Sheng et al. 2021 Sheng et al. https://creativecommons.org/licenses/by/4.0/ This content is distributed under the terms of the Creative Commons Attribution 4.0 International license.

The 6mA pattern change in starved *Tetrahymena* cells could be attributed to a combined effect of demethylation and methylation. The presence of active demethylation was confirmed by both SMRT sequencing and enzyme digestion-based analyses, which could contribute to the reduced 6mA level. Further identification and characterization of demethylase(s) will allow us to decipher their roles in starvation. Meanwhile, the changed activity of methyltransferases could fine-tune the 6mA composition. The starved cells can be considered an *AMT1* partial-loss-of-function strain (Table S2), wherein the expression levels of 6mA methyltransferase AMT1 and its potential partners AMT6/AMT7, specifically maintaining symmetric 6mA (5, 12), were downregulated. The impaired capability of AMT1 to conduct methyl addition on newly replicated DNA strands may leave some sites asymmetric after the completion of the last round of DNA replication before starvation. In contrast, the total removal of AMT1 in Δ*AMT1* cells almost abolished symmetric 6mA (12) (Table S2). Meanwhile, the expression level of another two MT-A70 family members, i.e., *AMT2* and *AMT5* (12, 17), was increased in starved cells, coinciding with the increased percentage of asymmetric 6mA. AMT2 and AMT5 are distinguished by several ZZ-type zinc fingers at the C terminus and grouped in the same subclade of eukaryotic MT-A70 MTases, distinct from AMT1 and its homologues. It is therefore tempting to hypothesize that AMT2 and AMT5 are specifically required for asymmetric methylation. Their functional division of labor awaits further investigation. Notably, the expression levels of *AMT2* and *AMT5* were not affected in Δ*AMT1* cells (Table S3), partially explaining the reduced site number of asymmetric 6mA (12) (Table S2).

10.1128/mSphere.01208-20.6 TABLE S2 Comparison of 6mA in vegetative WT (Veg), starved WT (S24), and vegetative Δ*AMT1* cells. Download Table S2, DOCX file, 0.02 MB. Copyright © 2021 Sheng et al. 2021 Sheng et al. https://creativecommons.org/licenses/by/4.0/ This content is distributed under the terms of the Creative Commons Attribution 4.0 International license.

10.1128/mSphere.01208-20.7 TABLE S3 Expression level change of methyltransferase genes in Δ*AMT1* (vegetative) and starved WT cells, normalized to levels in vegetative WT cells. Download Table S3, DOCX file, 0.02 MB. Copyright © 2021 Sheng et al. 2021 Sheng et al. https://creativecommons.org/licenses/by/4.0/ This content is distributed under the terms of the Creative Commons Attribution 4.0 International license.

In multicellular organisms, 6mA mainly distributes in intergenic regions and certain transposons (2, 7, 9, 10). In contrast to this, 6mA in unicellular eukaryotes such as *Tetrahymena* and *Chlamydomonas* is preferentially located in the 5′ region of protein-coding genes, in particular between nucleosomes (6, 8). In starved *Tetrahymena* cells, the degree of nucleosome positioning was increased. This could be partially explained by the fact that upon starvation two major nucleosome-perturbing processes, i.e., DNA replication and transcription, were (mostly) suspended (31) and highly reduced (39), respectively. Meanwhile, we found that asymmetric 6mA with high methylation level increased despite a global 6mA reduction in starved cells. It was previously reported in *Tetrahymena* that nucleosomes became fuzzier after the depletion of flanking 6mA (5, 12) and that 6mA dispersed outside linker DNA along with reduced nucleosome positioning (12), suggesting that 6mA and nucleosomes reinforce each other to establish the epigenetic landscape. Considering that *Tetrahymena* chromosomes were inferred to be maintained at an average of ∼45 copies (40), 6mA sites with high methylation levels indicated that more copies in this site are methylated among all 45 copies. These 6mAs would reinforce the nucleosome stacking along this site, stabilizing the nucleosome and raising its proportion among all nucleosomes located in this region. These assumptions were supported by the observation that the nucleosome positioning degree was higher for gain-of-6mA genes than loss-of-6mA genes. We therefore posit that the increased highly methylated asymmetric 6mA, together with the suspension of replication and reduction of transcription, accounts for the stronger nucleosome positioning in starved cells.

A large number of DEGs involved in metabolic adjustment (41), autophagic vacuole formation (36), the changed phosphorylation state of histone H1 (29), and the enhanced carbon and fatty acid metabolism to increase hormone levels (35) were detected. For these DEGs and starvation-responding genes, the 6mA amount change was positively associated, although weakly, with changes in their expression level, thereby linking 6mA with global transcription, which in turn accounts for the drastic phenotypic changes in starved cells. Other epigenetic factors, except for 6mA methylation, may also be involved in gene regulation during starvation.

Together, our results provide insights into how *Tetrahymena* fine-tunes its 6mA level and composition upon starvation, suggesting that a regulated 6mA response to environmental cues is evolutionarily conserved in eukaryotes.

## MATERIALS AND METHODS

### Cell culture.

Tetrahymena thermophila wild-type strain (SB210) was obtained from the *Tetrahymena* Stock Center (http://tetrahymena.vet.cornell.edu) and grown in super proteose peptone (SPP) medium at 30°C (42, 58). Cells at log phase (∼2 × 10^5^ cells/ml) were collected, washed, and starved in 10 mM Tris (pH 7.4). Cells were collected at 0, 3, 6, 12, 24, 48, and 72 h after the initiation of starvation (referred to as vegetative, S3, S6, S12, S24, S48, and S72).

### Immunofluorescence staining and imaging.

Vegetative and starved (S3, S6, S12, S24, S48, and S72) cells were collected for IF staining and imaging, which followed previously described procedures (8, 12).

### Preparation of *Tetrahymena* DNA and RNA samples.

Genomic DNA was extracted from the starved *Tetrahymena* cells (S24) (∼2 × 10^5^ cells/ml) using a Wizard genomic DNA purification kit (Promega; A1120). RNA samples were extracted from vegetative and starved cells (∼2 × 10^5^ cells/ml) with an RNeasy Plus minikit (Qiagen; 74134) (43). The quality and concentration of DNA and RNA samples were analyzed by agarose gel electrophoresis using a Qubit 3.0 fluorometer (Thermo Fisher Scientific).

### qRT-PCR.

Total RNA after DNase treatment (Invitrogen, AM1907) was reverse transcribed using an oligo(dT) primer and Moloney murine leukemia virus (M-MLV) reverse transcriptase (Invitrogen; 28025013) (44). For quantitative reverse transcription-PCR (qRT-PCR) analysis of gene expression levels in vegetative and starved cells, *SOR3* (TTHERM_00467390) was used for loading control and normalization. All PCR primers used in this study are listed in Table S4.

10.1128/mSphere.01208-20.8 TABLE S4 Primers used in this study. Download Table S4, XLSX file, 0.01 MB. Copyright © 2021 Sheng et al. 2021 Sheng et al. https://creativecommons.org/licenses/by/4.0/ This content is distributed under the terms of the Creative Commons Attribution 4.0 International license.

### DpnI/DpnII digestion and qPCR analysis.

The DpnI/DpnII digestion experiment followed previously described procedures (8). DpnI/DpnII-digested and nondigested DNAs (4 ng) were loaded into the qPCR analyzer using EvaGreen Express 2× qPCR master mix (low ROX) (ABM; MasterMix-LR). Primers flanking selected GATC sites are listed in Table S4. Primers in *SOR3* (TTHERM_00467390) were used as internal controls. Conversion sites are sites that were symmetric in vegetative cells but unmethylated in starved cells. The selected “retain site” is symmetrical in both vegetative and starved cells. The methylation status is reflected by fold difference between DpnI- and DpnII-digested samples in vegetative and starved cells (ΔΔCt = ΔCt_DpnI_ − ΔCt_DpnII_). ΔCt_DpnI_ and ΔCt_DpnII_ were normalized between digested and undigested samples, respectively (ΔCt_DpnI_ = Ct_DpnI_ − Ct_undigested_ and ΔCt_DpnII_ = Ct_DpnII_ − Ct_undigested_). The methylation status change was calculated as ΔΔΔCt = ΔΔCt_veg_ − ΔΔCt_S24_. As DpnI and DpnII cut methylated and unmethylated GATC sequences, respectively (45, 46), the fold difference in conversion sites between vegetative and starved cells should be larger than zero, while that of the retain site should be close to zero.

### UHPLC-QQQ-MS/MS analysis.

Vegetative and starved *Tetrahymena* cells were collected for ultra-high-performance liquid chromatography–triple-quadrupole tandem mass spectrometry (UHPLC-QQQ-MS/MS) analysis, which followed previously described procedures (8, 12).

### SMRT data analysis.

Genomic DNA prepared for SMRT sequencing library was extracted from starved cells (S24) (SB210) using a Wizard genomic DNA purification kit (Promega; A1120), and sequencing was carried out by Novogene Co. Ltd. (Beijing, China). Even though SMRT sequencing does not discriminate 6mA versus 1mA, we could call 6mA with confidence from SMRT sequencing results, as 1mA was not previously detected in the *Tetrahymena* genome by mass spectrometry (12).

The latest SB210 MAC genome downloaded from the *Tetrahymena* genome database (TGD) (http://ciliate.org) (47) was used as the reference for read mapping. 6mA was identified using the Base Modification and Motif Analysis protocol with default parameters in SMRT Link v5.10 (Pacific Biosciences). Considering the different sequencing depths between vegetative and starved cells, all data were normalized to 100× while using a strict cutoff (Qv > 30 and coverage > 25×) to filter out unauthentic modifications.

To calculate 6mA density across chromosomes, smooth curves were plotted by ggplot2 in R (48). For composite analysis and motif identification, 6mA was divided into groups based on their methylation level (L1, 0 to 20%; L2, 20 to 40%; L3, 40 to 60%; L4, 60 to 80%; L5, 80 to 100%) or motifs (symmetric/asymmetric/non-AT). The number and percentage of sites of different 6mA groups were calculated by customized Perl scripts and plotted using GraphPad Prism 6 (49).

The genome-wide distribution of 6mA groups on chromosomes was counted by customized Perl scripts. The 6mA density was calculated as the number of methylated adenine sites divided by the total number of adenine sites (6mA/A) in each bin (bin size = 1 kb).

For analysis of 6mA distribution among genes, 18,914 long (>1 kb) genes were selected. The gene body length was scaled to 1 unit, and length was extended by 1 unit on each side. Customized Perl scripts were used for locus statistics (bin size = 0.05). To calculate the distribution of 6mA around the TSS, the number of 6mA sites was accumulated in every base from 1,000 nucleotides (nt) upstream to 2,000 nt downstream of the TSS. The 6mA amount was defined as the number of methylated adenine sites combined with their methylation level.

To identify conserved motifs around the methylated adenines, sequences between 20 nt upstream and 20 nt downstream of 6mA sites were extracted. Local motifs nearby 6mA were illustrated by WebLogo3 (50) and GraphPad Prism 6.

To determine the correlation between 6mA amount change (S24 − Veg) in the 1 kb downstream of TSS and their expression level change (log_2_ fold change) in genes, Pearson correlation analysis was carried out by SPSS v. 22.0. (51). Raw values of the 6mA amount change and gene expression level change were used in this analysis. The positive correlation between 6mA amount change and gene expression level change suggested that genes with increased 6mA level (6mA amount change increase) tend to be upregulated (log_2_ fold change increase) upon starvation. Starvation-responding genes included starvation-induced genes (total counts: Veg < 300, S24 > 1,500) and starvation-repressed genes (total counts: Veg > 1,500, S24 < 300), which were defined according to their numbers of reads in vegetative and starved cells. Highly regulated genes were defined as the top 10% genes with different expression levels in vegetative and starved cells.

### RNA sequencing and data analysis.

A total of six RNA samples of T. thermophila were sequenced, three replicates each for vegetative (Veg) and starved cells (S24), respectively. After trimming of sequencing adapters and filtering of low-quality reads according to Trimmomatic (52) (TruSeq3-PE.fa, 2,30,10; leading, 3; trailing, 3; sliding window, 4,15; minlen, 80), the numbers of reads mapped to the genome were determined using HISAT2 software (53). FeatureCounts (54) was implemented for counting reads to genomic features with the assembled transcripts as a reference. Effective expression levels (number of fragments per kilobase per million reads [FPKM] > 1) were calculated with DEseq2 based on RNA-Seq coverage of these strains for counting the Pearson’s correlation coefficients of gene expression (55). DEGs were also identified by DEsq2 (log_2_ fold change > 2 or < −2; *P* < 0.05). Pathway analysis of DEGs was carried out on the KEGG web server (https://www.genome.jp/kegg/pathway.html) (56).

### Nucleus purification and MNase sequencing analysis.

Nucleus purification was carried out following established protocols (57). Approximately 5 × 10^7^ purified MACs from vegetative and starved cells (S24) were digested with micrococcal nuclease (MNase; 400 U/ml; New England Biolabs [NEB]; M0247S) at 25°C for 15 min. Mononucleosome-sized DNA was collected by phenol-chloroform extraction for sequencing.

Sequencing reads were mapped to the latest MAC genome assembly in TGD (http://ciliate.org) (47) and analyzed following procedures described before (12).

### Data availability.

All sequencing data have been deposited in the NCBI database under accession number PRJNA545568.
