# Supplementary figures and images for: Case Study of the Response of N6-Methyladenine DNA Modification to Environmental Stressors in the Unicellular Eukaryote Tetrahymena thermophila
Source: mSphere. 2021 May 28;6(3):e01208-20. doi: 10.1128/mSphere.01208-20 (PMC8265677; doi:10.1128/mSphere.01208-20)

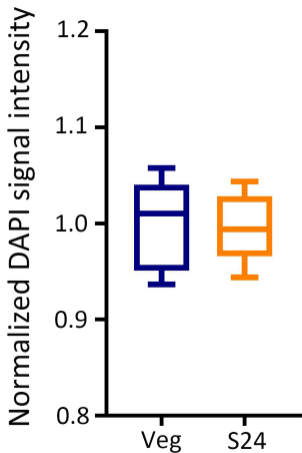

Supplement: FIG S1 [file msphere.01208-20-sf001.pdf]

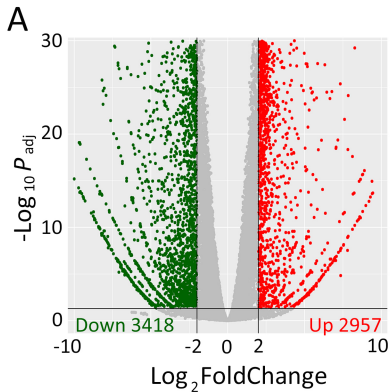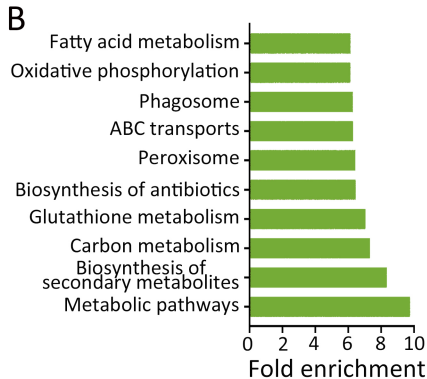

Supplement: FIG S2 [file msphere.01208-20-sf002.pdf]

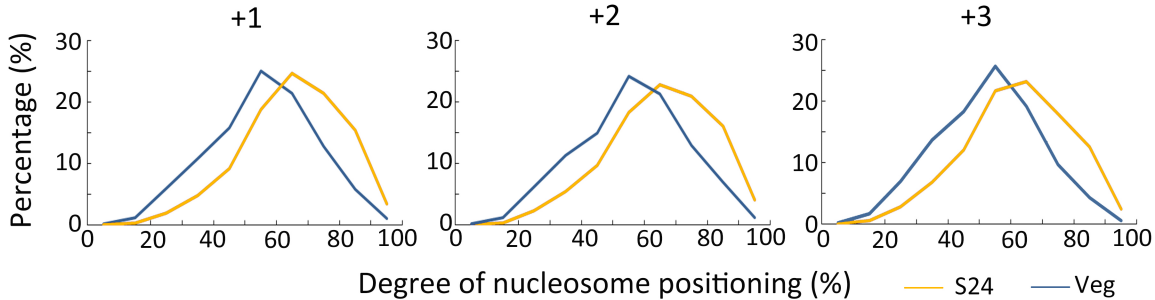

Supplement: FIG S3 [file msphere.01208-20-sf003.pdf]

## Mass spectrometry

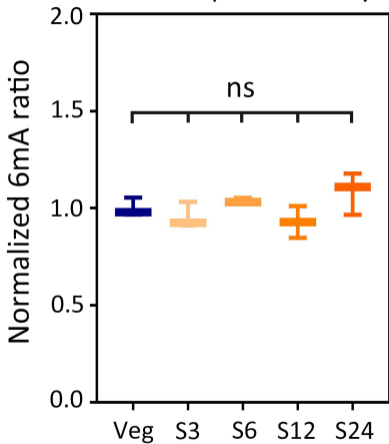

Supplement: FIG S4 [file msphere.01208-20-sf004.pdf]
