# Supplementary material for: Case Study of the Response of N6-Methyladenine DNA Modification to Environmental Stressors in the Unicellular Eukaryote Tetrahymena thermophila
Source: mSphere. 2021 May 28;6(3):e01208-20. doi: 10.1128/mSphere.01208-20 (PMC8265677; doi:10.1128/mSphere.01208-20)
Supplement: TABLE S1 [file msphere.01208-20-st001.docx]

**Table S1.** SMRT Sequencing data quality control.

| **Job Metric** | **Veg** | **S24** |
| --- | --- | --- |
| Adapter Dimers (0-10bp) | 0.06% | 0.04% |
| Short Inserts (11-100bp) | 1.00% | 0.89% |
| Number of Bases | 18,729,989,358 | 12,301,514,768 |
| Number of Reads | 1,684,630 | 1,077,887 |
| N50 Read Length | 18,676 | 18,223 |
| Mean Read length | 11,118 | 11,299 |
| Mapped Reads | 1,311,395 | 948,914 |
| Mapped Number of Read Bases | 14,919,325,920 | 10,726,472,457 |
| Mapped Mean Concordance | 84.19% | 86.0% |
| Average Reference Length | 570,971 | 570,971 |
| Average Reference Coverage | 137.49 | 103.79 |
| Longest Reference Contig | chr_179 | chr_179 |
