# Supplementary material for: Case Study of the Response of N6-Methyladenine DNA Modification to Environmental Stressors in the Unicellular Eukaryote Tetrahymena thermophila
Source: mSphere. 2021 May 28;6(3):e01208-20. doi: 10.1128/mSphere.01208-20 (PMC8265677; doi:10.1128/mSphere.01208-20)
Supplement: TABLE S2 [file msphere.01208-20-st002.docx]

**Table S2.** Comparison of 6mA in vegetative WT (Veg), starved WT (S24), and vegetative Δ*AMT1* cells.

|  | **Veg** | | **S24** | | | **Δ*AMT1*** | | |
| --- | --- | --- | --- | --- | --- | --- | --- | --- |
|  | Sites | Percentage (%) | | Sites | Percentage (%) | | Sites | Percentage (%) |
| 6mA density (6mA/A) | - | 0.54 | | - | 0.39 | |  | 0.16 |
| Methylated adenines | 436276 | - | | 312521 | - | | 127083 | - |
| Symmetric-6mA | 133199‬*2 | 61.1 | | 78507*2 | 50.3 | | 862*2 | 1.4 |
| Asymmetric-6mA | 116465 | 26.7 | | 137926 | 44.0 | | 57176 | 45.0 |
| Non-ApT-6mA | 53413 | 12.2 | | 17581 | 5.6 | | 68183 | 53.7 |
| L1 (0-20%) | 1214 | 0.3 | | 359 | 0.1 | | 2340 | 1.8 |
| L2 (20-84%) | 14649 | 3.4 | | 2459 | 0.8 | | 26403 | 20.8 |
| L3 (40-60%) | 66749 | 15.3 | | 19300 | 6.2 | | 53113 | 41.8 |
| L4 (60-80%) | 191161 | 43.8 | | 116167 | 37.2 | | 35966 | 28.3 |
| L5 (80-100%) | 162503 | 37.2 | | 174236 | 55.8 | | 9261 | 7.3 |
| Mass spectrometry (6mA/A) | - | 100% | | - | 108% | | - | 22% |
