# Supplementary material for: Case Study of the Response of N6-Methyladenine DNA Modification to Environmental Stressors in the Unicellular Eukaryote Tetrahymena thermophila
Source: mSphere. 2021 May 28;6(3):e01208-20. doi: 10.1128/mSphere.01208-20 (PMC8265677; doi:10.1128/mSphere.01208-20)
Supplement: TABLE S3 [file msphere.01208-20-st003.docx]

**Table S3.** Expression level change of methyltransferase genes in Δ*AMT1* (vegetative) and starved WT cells, normalized by vegetative WT cells.

| **Gene name** | **TTHERM No.** | **Δ*AMT1*** | **S24** | |
| --- | --- | --- | --- | --- |
|  |  | RNA-Seq | RNA-Seq | qRT-PCR |
| *AMT1* | 00704040 | - | -2.57 | -1.15 |
| *AMT2* | 00388490 | 0.25 | 0.20 | 0.68 |
| *AMT5* | 00136470 | -0.04 | 0.64 | 3.03 |
| *AMT6* | 01005150 | 0.06 | -4.02 | -2.68 |
| *AMT7* | 00301770 | 0.11 | -1.24 | 0.08 |
